# Epidemiology of depression in schizophrenia patients living in Africa: a systematic review and meta-analysis

**DOI:** 10.1192/j.eurpsy.2023.600

**Published:** 2023-07-19

**Authors:** F. T. Endomba, M. Tariku

**Affiliations:** ^1^University of Burgundy, Dijon, France; ^2^Haramaya University, Haramaya, Ethiopia

## Abstract

**Introduction:**

Various comorbid conditions can worsen the morbidity and mortality of schizophrenia, and this is the case for depression, especially through suicidal behaviors and cardiometabolic impairments. There is a scarcity of summarizing data on depressive symptoms and disorders among schizophrenia patients living in Africa.

**Objectives:**

The aim of this meta-analytic review was to estimate the prevalence of depression in people living with schizophrenia in Africa.

**Methods:**

We systematically searched for relevant articles published from inception to July 05, 2022, in PubMed/MEDLINE, EMBASE, and African Journals Online. We appraised the risk of bias using the Joanna Briggs Institute (JBI) Critical Appraisal Checklist for studies reporting prevalence data, and estimated the pooled prevalence of depression among patients with schizophrenia using a random-effects meta-analytic model. We performed meta-regression and subgroup analyses to assess potential mediators of our estimates. We based the report of our findings on the Preferred Reporting Items for Systematic Reviews and Meta-analyses guidelines (2020). We registered our protocol in PROSPERO (CRD42022315717).

**Results:**

From 791 initial records, 10 studies were finally included in our qualitative and quantitative syntheses (Figure 1). These studies encompassed 2265 patients with schizophrenia (male-to-female ratio = 1.94), and were conducted between 2001 and 2019, in Egypt (n = 1/10), Ethiopia (n = 4/10), Morocco (n = 1/10), Nigeria (n = 1/10), South Africa (n = 2/10), and Tunisia (n = 1/10). The mean age of participants ranged from 33.8 to 49.2 years, and the most used tool was the Calgary Depression Scale for Schizophrenia (n = 4/10). The pooled prevalence rate of depression was 23.93% (95% CI: 19.43% – 28.73%), with substantial heterogeneity (*I*² = 84%). The prevalence of depression significantly varied according to screening tool used. The frequencies for Northern and Sub-Saharan Africa were respectively 31.9% (95% CI: 24.8% – 39.5%) and 21.1% (95% CI: 16.7% – 25.9%), with a significant difference between these subgroups (Figure 2). A higher prevalence of depression was associated with a lower percentage of schizophrenia patients with high education levels. Among schizophrenia people with depression (n = 250), 46.04% (95% CI: 30.07% – 62.42%) reported past or current suicide behaviors. The risk of bias was low for three studies, moderate for two studies, and high for five studies. The certainty was very low, and we found no publication bias.

**Image:**

**Figure:**
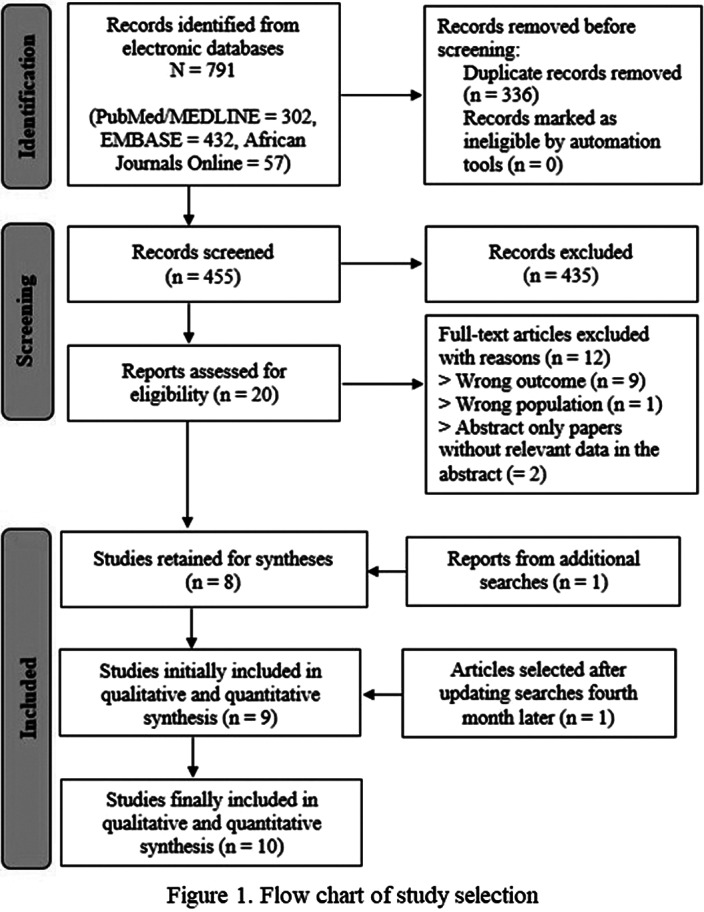

**Image 2:**

**Figure:**
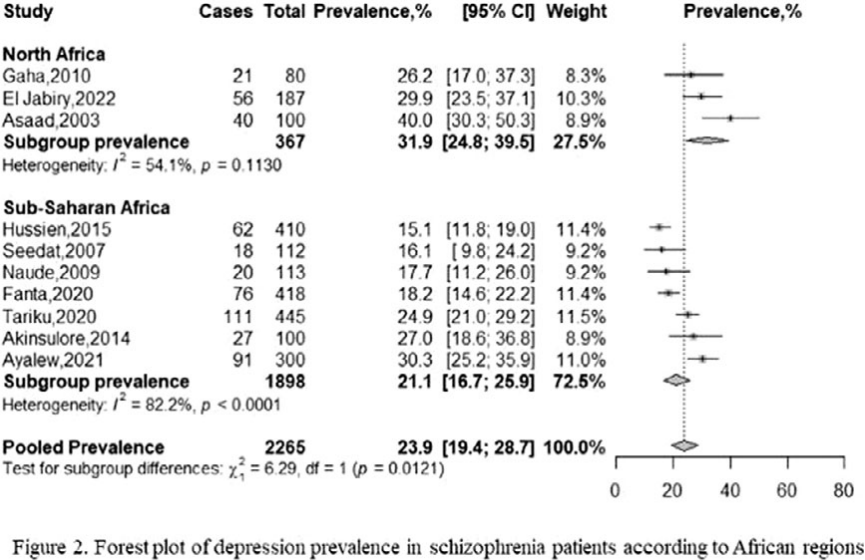

**Conclusions:**

Approximately one in every four schizophrenia patients living in Africa was positively screened for depression. This review draws health professionals’ attention caring people with schizophrenia, and calls for further studies with a harmonization of screening tool, a better representativity of some subregions, and the assessment of key potential factors such as perceived stigma and self-stigma.

**Disclosure of Interest:**

None Declared

